# Phylogenomic Relationships and Evolution of Polyploid Salix Species Revealed by RAD Sequencing Data

**DOI:** 10.3389/fpls.2020.01077

**Published:** 2020-07-17

**Authors:** Natascha D. Wagner, Li He, Elvira Hörandl

**Affiliations:** ^1^ Department of Systematics, Biodiversity and Evolution of Plants (with Herbarium), University of Goettingen, Göttingen, Germany; ^2^ College of Biological Sciences and Technology, Beijing Forestry University, Beijing, China

**Keywords:** phylogenomics, polyploidy, restriction associated DNA sequencing, shrub willows, SNiPloid

## Abstract

Polyploidy is common in the genus *Salix*. However, little is known about the origin, parentage and genomic composition of polyploid species because of a lack of suitable molecular markers and analysis tools. We established a phylogenomic framework including species of all described sections of Eurasian shrub willows. We analyzed the genomic composition of seven polyploid willow species in comparison to putative diploid parental species to draw conclusions on their origin and the effects of backcrossing and post-origin evolution. We applied recently developed programs like SNAPP, HyDe, and SNiPloid to establish a bioinformatic pipeline for unravelling the complexity of polyploid genomes. RAD sequencing revealed 23,393 loci and 320,010 high quality SNPs for the analysis of relationships of 35 species of Eurasian shrub willows (*Salix* subg. *Chamaetia/Vetrix*). Polyploid willow species appear to be predominantly of allopolyploid origin. More ancient allopolyploidization events were observed for two hexaploid and one octoploid species, while our data suggested a more recent allopolyploid origin for the included tetraploids and identified putative parental taxa. SNiPloid analyses disentangled the different genomic signatures resulting from hybrid origin, backcrossing, and secondary post-origin evolution in the polyploid species. Our RAD sequencing data demonstrate that willow genomes are shaped by ancient and recent reticulate evolution, polyploidization, and post-origin divergence of species.

## Introduction

Although the impact of polyploidy in plant evolution is evident (Soltis and Soltis, 2009; Wood et al., 2009; Jiao et al., 2011; Mayrose et al., 2011; Barker et al., 2016), there is much to discover about the impact of polyploidy on evolution of plant genomes. Polyploids may originate from combining genomes via hybridization (allopolyploids) or from intraspecific genome duplication (autopolyploids). Post-origin evolution and introgression may further leave genomic signatures (Peralta et al., 2013). This information, however, is crucial to understand the evolution of species. The potential offered by the rise of high-throughput sequencing tools to analyze the origin of natural polyploid plant species has not yet been fully exploited. The main reasons might be the difficulties in variant calling, especially the need to distinguish different alleles derived from the same parent (homologs) from alleles originating from different parents (homeologs; Clevenger et al., 2015). Other difficulties are the necessity to filter paralogs (Hirsch and Buell, 2013), the uncertainty of allelic dosage as well as the lack of appropriate bioinformatic tools and models (Meirmans et al., 2018). Given a sufficient coverage, high-throughput sequencing methods are generally suited to detect all different alleles at a specific locus. However, to use a massive amount of short read data for the analysis of polyploids is still challenging, especially, if no reference genome is available.

Restriction Associated DNA (RAD) sequencing (Baird et al., 2008) is a widely used method that can harvest ten-thousands of informative SNPs representing the whole genome. The huge amount of biallelic SNPs is well suited to study evolutionary processes, resolve reticulate relationships, reconstruct species trees, and to analyze polyploid species in a phylogenetic framework (Bryant et al., 2012; Dufresne et al., 2014; Clevenger et al., 2015; Clevenger et al., 2018). However, there are only few studies using RAD sequencing data when dealing with polyploid non-model plants (Chen et al., 2013; Mastretta-Yanes et al., 2014; Qi et al., 2015; Brandrud et al., 2020; He et al., 2020).

The genus *Salix* L. (Salicaceae) comprises about 400-450 species of trees and shrubs that are mainly distributed in the Northern Hemisphere. About two thirds of the species occur in Eurasia, while about 140 species are distributed in America. Classical taxonomy and systematics in *Salix* have proven to be extremely difficult because of dioecy, simple, reduced flowers, common natural formation of hybrids, high intraspecific phenotypic variation, and the presence of polyploid species (Skvortsov, 1999; Hörandl et al., 2012; Cronk et al., 2015). Phylogenetic analyses using traditional Sanger sequencing and standard markers (like ITS) were able to separate subgenus *Salix* from a big clade uniting the two subgenera *Chamaetia* and *Vetrix*, but lacked any interspecific resolution (Leskinen and Alström-Rapaport, 1999; Chen et al., 2010; Percy et al., 2014; Lauron-Moreau et al., 2015; Wu et al., 2015). Especially in the case of polyploidization and frequent hybridization, the usage of uniparentally inherited plastid markers is useful for phylogenetic reconstruction. However, plastid markers show only little variation within willows (see Leskinen and Alstrom-Rapaport, 1999; Chen et al., 2010; Savage and Cavender-Bares, 2012; Percy et al., 2014; Lauron-Moreau et al., 2015; Wu et al., 2015). More recently, the complete plastomes of twelve *Salix* species were reconstructed and showed only 0.9% variability (N.D.Wagner, unpublished data). The *Chamaetia/Vetrix* clade comprises three quarters of species diversity in *Salix* ranging from creeping arctic-alpine dwarf shrubs to medium-sized trees (Wagner et al., 2018). In contrast to many other woody plant lineages about 40% of the species are polyploid (Suda and Argus, 1968), ranging from diploid to octoploid species (rarely deca-/dodecaploid), based on the chromosome number *x* = 19 (Argus, 1997). Previous studies proposed that ancient genome duplications occurred several times in *Salix* (Dorn, 1976; Leskinen and Alström-Rapaport, 1999) and in the Salicoid lineage (Tuskan et al., 2006). Paleopolyploid species are more difficult to recognize, because their cytological behaviour has undergone ‘diploidization’, i.e. they have returned to regular chromosome pairing at meiosis as typical for diploid sexual species (Comai, 2005). Cold climatic regimes during the Pleistocene may have triggered spontaneous autopolyploidization by unreduced gamete formation (Ramsey and Schemske, 1998). Recent allopolyploidization (‘young polyploidy’) may have happened during the Pleistocene, when climatic oscillations caused range fluctuations of plant species and enhanced secondary contact hybridization of previously isolated species (Hewitt, 2004; Abbott et al., 2013; Kadereit, 2015). While homoploid hybridization may result in introgressive hybridization with the parental species (Hardig et al., 2010; Gramlich and Hörandl, 2016; Gramlich et al., 2018), allopolyploidy can establish a rapid crossing barrier against the parents and hence may result in the evolution of an independent lineage. In willows, natural hybridization is a frequent phenomenon (Argus, 1997; Skvortsov, 1999; Hörandl et al., 2012) and may occur even between distantly related species (e.g. Hardig et al., 2000; Gramlich et al., 2018). While in the 19^th^ century experimental crosses in *Salix* were popular and even triple or multiple hybrids have been produced (Wichura, 1865), the origin of most natural polyploid species is still unknown. Population genetic markers like SSRs and AFLPs have been used so far to study polyploids (Barcaccia et al., 2014; Guo et al., 2016), but in the *Chamaetia/Vetrix* clade the polyploid species have never been analyzed with molecular markers. We expect to trace signatures of ancient and/or recent hybrid origin, secondary lineage-specific evolution, backcrossing, and introgression in their genomes.

RAD sequencing (Baird et al., 2008) was recently used to overcome the lack of phylogenetic information within the genus *Salix* (Gramlich et al., 2018; Wagner et al., 2018; He et al., 2020). Wagner et al. (2018) published the first well-resolved phylogeny of diploid European members of the *Chamaetia*/*Vetrix* clade, while He et al. (2020) recognized a recent adaptive radiation of willows in the Hengduan mountains. Gramlich et al. (2018) successfully used RAD sequencing to analyze recent homoploid hybridization patterns and introgression between two *Salix* species on alpine glacier forefields. RAD loci in *Salix* represent almost exclusively the nuclear genome, and provide 10-thousands of SNPs from both conservative and rapidly evolving non-coding genomic regions (Gramlich et al., 2018). Therefore, this method has the potential to resolve reticulate relationships and to analyze the origin of the polyploid species in *Salix.*

In this study we want to test the utility of RAD sequencing data for the analysis of polyploid willow species. We will use a comprehensive approach of different traditional and recently developed analytical tools to elucidate the evolutionary scenarios of polyploid origin in Eurasian shrub willows. By using various approaches, we will 1) establish a phylogenomic framework of the *Vetrix/Chamaetia* clade, 2) test for recent allopolyploidization by hybridization of two (or more) related diploid parents versus more ancient allopolyploidization of distantly related progenitors of extant species, and 3) test for autopolyploidization. Based on this information, we 4) try to get insights into post-origin evolution of polyploid species by analyzing their genomic composition.

## Material and Methods

### Sampling

For this study, we sampled 28 diploid species (incl. two subspecies), one triploid, three tetraploid, two hexaploid and one octoploid species representing 19 sections sensu Skvortsov (1999) of the *Chamaetia*/*Vetrix* clade. *Salix triandra* (subg. *Salix*) was included to serve as outgroup (Wu et al., 2015). Hence, the complete sample set consists of 36 *Salix* species. The samples were collected in Central and Northern Europe as well as in China and determined after Fang et al. (1999); Skvortsov (1999) and Hörandl et al. (2012). The diploid species represent all main European lineages (Rechinger and Akeroyd, 1993). Leaves were dried in silica gel and herbarium voucher specimens were deposited in the herbarium of the University of Goettingen (GOET). Two to five accessions per species were included in the analyses given a total of 133 samples (**Supplement Table S1**).

### Ploidy Determination Using Flow Cytometry

The ploidy levels of seven species (23 samples) with unknown chromosome counts were estimated using flow cytometric analyses based on DAPI (4’, 6-diamidino-2-phenylindole) fluorochrome applying a modified protocol of Suda and Trávníček (2006) as described in He et al. (2020). *Salix caprea* with known ploidy level (2x=2n=38) was used as an external standard.

### Molecular Treatment and Analyses

The DNA of all samples was extracted using the Qiagen DNeasy Plant Mini Kit following the manufacturer´s instructions (Valencia, CA). After quality check, the DNA was sent to Floragenex, Inc. (Portland, Ore., USA) where the sequencing library preparation was conducted after Baird et al. (2008) using the restriction enzyme *Pst*I (see Wagner et al., 2018 for details). Polyploids require an increased depth of coverage based on the bigger genome size and the higher number of alleles (Hirsch and Buell, 2013; Clevenger et al., 2015). Thus, we sequenced polyploid and diploid taxa on different plates to avoid loss of coverage.

There are several tools available to analyse RAD sequencing data. While e.g. STACKS (Catchen et al., 2013) is more suited to population genetic analyses, ipyrad is predominantly used for phylogenetic approaches (see Eaton et al., 2017). Ipyrad conducts quality filtering and de novo locus identification and genotyping, with the advantage that it can handle insertion-deletion variation among alleles, and it is therefore better suited for studies of a broader taxonomic scale (Andrews et al., 2016). The quality of the resulting single-end 100bp long sequence reads was checked using FastQC v.0.10.1 (Andrews, 2010). After de-multiplexing, the reads were used to run ipyrad v.0.7.28 (Eaton and Overcast, 2020) with a clustering threshold of 85% and a minimum depth of eight reads for base calling. The clustering was done with VSEARCH as implemented in ipyrad v.0.7.28. The maximum number of SNPs per locus was set to 20, the maximum number of indels to 8. We set a threshold of maximal four alleles per site in the final cluster filtering. Ipyrad summarizes the underlying allelic information into a consensus sequence in the form of ambiguous sites at heterozygous positions, which is a common strategy to face the problem of more than two alleles (Blischak et al., 2018b). The resulting sequence information can be used for downstream analyses. Initial settings for the minimum number of samples sharing a locus (m) ranged from m4 (average number of accessions per species) to m120 (loci present in 90% of accessions) and were optimized as described in Wagner et al. (2018). Statistics for the tested settings are summarized in **Supplement Table S2**. The m40 dataset (29.06% missing data) was used for phylogenetic analyses and the m100 dataset (9.19% missing data) for the genetic structure analysis. Additionally, we performed tree reconstruction with ipyrad for each clade separately that contains one or more polyploid samples applying the same settings.

We inferred phylogenetic relationships on concatenated alignments of the complete dataset as well as for each clade separately by using the GTR+ Γ model of nucleotide substitution implemented in RAxML v.8.2.4 (Stamatakis, 2014). We conducted for each ML analysis a rapid bootstrapping (BS) analysis with 100 replicates. Next to BS we applied *quartetsampling* (QS; Pease et al., 2018) with default settings to test statistical support of a given topology. We ran 300 replicates using the –L option (minimum likelihood differential). QS is able to distinguish between conflicting signals and poor phylogenetic information. The Quartet Concordance score [QC, (1,-1)] gives the support of the current topology, the Quartet Differential score [QD, (0,1)] is an indicator of introgression and the Quartet Informativeness score [QI, (0,1)] quantifies the informativeness of each branch. For each phylogeny shown here, the observed QS values (QC/QD/QI) were visualized along with the BS values above and below branches, respectively.

### Species Tree Estimation Using SNAPP

The SNAPP method (Bryant et al., 2012) estimates species trees directly from biallelic markers (e.g., SNP data), and bypasses the necessity of sampling the gene trees at each locus. This is specifically advantageous for RAD sequencing data sets based on short loci. For species tree calculation with SNAPP v1.4.2 we used the unlinked SNPs output (one SNP per locus, uSNPs) of the clade-specific ipyrad pipeline. We created the input file using BEAUti v2.5.1 (Bouckaert et al., 2014). The multiple accessions of a species were assigned to one taxon. As priors we used default settings for speciation rate (lambda of 1/X). Non-polymorphic sites were excluded. We ran the analysis in BEAST2 (Bouckaert et al., 2014). The number of mcmc generations was up to 10,000,000 and we sampled every thousandth tree. The results were checked with TRACER 1.7.1 (Rambaut et al., 2018). The resulting trees were summarized in the SNAPP tree analyzer tool. The trees of all RAxML and SNAPP analyses were obtained in FigTree v1.4.3 (Rambaut, 2014).

### Genetic Structure and Network Analyses

To test for an influence of reticulate evolution on the genetic composition of the included species, we explored the genetic structure of each sample. We simplified the data to be able to combine diploids and polyploids in the same STRUCTURE analyses by summarizing the observed allelic information of a polyploid to a “diploid” consensus sequence (e.g. the four alleles “AATT” will be reduced to “AT”). We used the uSNPs of the m100 data set (< 10% missing data) for the complete dataset to avoid bias by too much missing data. The clade specific analyses were performed using the uSNP data of the clade specific ipyrad runs. We chose a burn-in of 10,000 and a MCMC of 100,000 replicates, with three iterations of each value of K (K=number of genotypic groups). After an initial test, the range of K was set from 2 to 7 in the final analysis. For the subclades, K was set from two up to the number of taxa included. The optimal K value was estimated by inferring the Evanno test revealing the optimal delta K value in Structure Harvester (Earl and vonHoldt, 2012).

Reticulate relationships resulting from hybridization or allopolyploidy are not well represented by bifurcating tree topologies (Huson and Bryant, 2006; McBreen and Lockhart, 2006). To overcome this problem, we utilized SplitsTree4 (Huson and Bryant, 2006) in order to reconstruct possible network-like evolutionary relationships among the species for each clade. Based on informative SNP data (see Feng et al., 2018), we generated the split network by implementing NeighbourNet analysis with variance of ordinary least squares complemented by a bootstrapping with 1000 replicates to test for statistical support. Missing data were treated as unknown in all analyses.

### Hybridization Detection

HyDe (Hybridization Detection; Blischak et al., 2018a) allows testing for hybridization and introgression at a population or species level based on D-Statistics by estimating the amount of admixture (γ). We applied the “run_hyde_mp.py” script to test for putative parent-hybrid combinations with respect to the polyploid individuals. HyDe uses p-values to test for the significance of results. While a 50:50 hybrid is characterized by a γ-value of about 0.5, very low levels of admixture (e.g. 0.01 = close to parent P1; 0.99 = close to parent P2) may be indicators for several processes such as ILS and more ancient hybridization. After initial tests, we used a range of γ=0.4‑0.6 to identify recent hybridization events in the data set, and intermediate ranges of γ= 0.1‑0.4 and 0.6‑0.9 for older events. We excluded significant values <0.1 and >0.9. The complete sequences as well as the uSNP data of the clade-specific analyses were used as input data, as the authors stated this datatype as the “most appropriate input” (Blischak et al., 2018a). We tested two approaches: first, we assigned all individuals of a species to one taxon, and second, we tested all individuals as separate entities. The latter approach is better to detect individual admixture. To underline the usage of HyDe in RAD sequencing data we performed a preliminary test on six diploid naturally formed F1 hybrids between *S. helvetica* and *S. purpurea* (Gramlich et al., 2018). The results revealed γ-values of 0.4‑0.5 (complete sequence data) and 0.3‑0.4 (uSNPs). These values are in accordance with recent studies which showed similar γ-values for modelled and empirical hybrid data (Blischak et al., 2018a; Zhang et al., 2019).

### Categorization of SNPs Using the SNiPloid Pipeline

SNiPloid (Peralta et al., 2013) is a tool developed to analyze the SNP composition of recently established allotetraploid taxa emerging from diploid parental lineages. Originally, this approach is based on RNA sequencing data. By mapping the sequencing reads of an allotetraploid individual to a reference (diploid parent 1) and comparing the observed SNPs and read depth with the results of diploid parent 2, conclusions on the SNP composition of the tetraploid species can be drawn. SNiPloid is able to distinguish five different SNP categories (Peralta et al., 2013, **Figure 5A**). Category 1/2 = “inter-specific” SNPs describes that the observed allele in the polyploid is identical with only one of the parental species. Category 3/4 = “derived” SNPs is attributed, when the variation observed in the tetraploid is not identified between parental genomes. This hints to a mutation that occurred after the polyploidization event. Finally, category 5 corresponds to putative homeo-SNPs, i.e., the tetraploid is heterozygous for homeologous alleles of both parental genomes (**Figure 5A**). SNPs that do not fall into the categories mentioned above are categorized as “other”.

**Figure 1:**
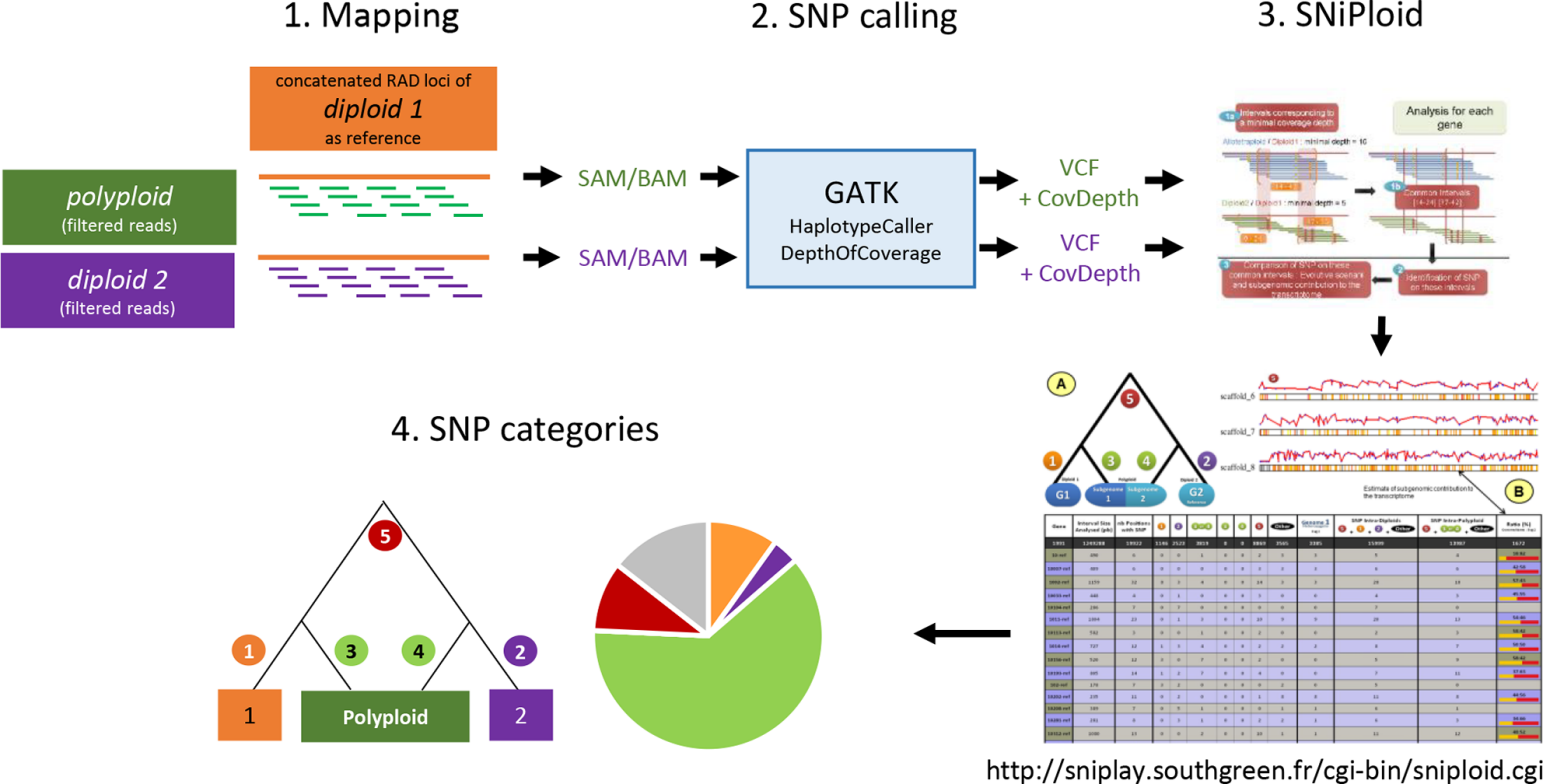
SNiPloid workflow adapted from (Peralta et al., 2013). The filtered RAD sequencing reads of the polyploid and one putative parental species (*Diploid 2*) are mapped to the concatenated RAD loci of the second putative parental species (*Diploid 1*) that act as a “pseudo-reference”. SNPs and coverage depth are determined with GATK tools “HaplotypeCaller” and “DepthOfCoverage”. The resulting “VCF” and “CovDepth” files of both samples serve as input for SNiPloid. The output of SNiPloid contain the assigned SNP categories 1‑5 for each compared SNP. Those data are finally summarized in a pie chart that shows the proportions of the observed categories.

To use the advantages of this pipeline and to endeavour the SNP composition of the tetraploids in our dataset we adapted the SNiPloid pipeline to RAD sequencing data (**Figure 1**). For our study, we used the concatenated consensus RAD loci of one putative diploid parental species as pseudoreference (similar as in Chen et al., 2013) for each test. The obtained RAD loci for every single individual (SAMPLE.consens file) were transferred into FASTA format. The indexing of the reference was done in BWA/0.7.12 (Li and Durbin, 2009). The sequence dictionary was created with PICARD/2.10.5 using the CreateSequenceDictionary tool (http://broadinstitute.github.io/picard/). The trimmed and filtered reads of the tetraploid species as well as of the second putative diploid parental species were mapped to the indexed reference applying the BWA/0.7.12 mapping tool using the MEM algorithm. The option -M (mark shorter split hits) for compatibility with PICARD was used, and 12 threads were applied for calculations (-t 12). The PICARD suite was used to sort the SAM file and to add read groups to the mapped alignment (‘AddOrReplaceReadGroups’). The resulting BAM file was indexed with SAMTOOLS v. 1.8 (Li et al., 2009) and used as input for the Genome Analysis Toolkit [GATK 3.8; McKenna et al. (2010)] to analyze the read depth (“DepthOfCoverage”) and the observed SNPs (“HaplotypeCaller”). For the tetraploid samples the—ploidy argument of HaplotypeCaller was set to 4. The generated read depth and the vcf files were eventually used as input for the executable perl script SNiPloid.pl (http://sniplay.southgreen.fr/cgi-bin/sniploid.cgi). The results of each tested combination were summarized in a table and pie diagram, given the percentage of each SNP category, and were then compared to each other. Recently established allotetraploid hybrids should show a high proportion of homeo-SNPs, because they should contain half of the alleles of diploid “parent1” and the other half of diploid “parent2” and should therefore be heterozygous for most loci. With ongoing speciation, we assume a higher proportion of cat 3/4 SNPs that are specific for the polyploid and originated after the polyploidization event. Backcrossing with one or both parents might lead to an increase of interspecific SNPs (cat 1/2), but these shared SNPs might also be the result of incomplete lineage sorting. The remaining “other” SNPs contain all other SNP combinations that cannot be assigned to any of the defined categories. This includes, e.g., SNPs that are heterozygous in the parental taxa and SNPs that show more than two alleles. They partly represent shared ancient polymorphisms of the whole group.

We initially narrowed down the number of combinations by choosing the most likely putative parental species/lineages according to the results of RAxML, SNAPP, NeighbourNet, and STRUCTURE. These parental combinations were tested with SNiPloid (see **Table 1**).

**Table 1:** Allotetraploid species and their putative diploid parental species according to results of the phylogeny and the combination of NeighborNet, HyDe, STRUCTURE, and SNiPloid analyses.

Polyploid	Parent 1	Parent 2
*Salix caesia (4x)*	*S. purpurea*	*S. repens*
*Salix cinerea (4x)*	*S. aurita*	*S. appendiculata*
*Salix laggeri (4x)*	*S. appendiculata*	*S. caprea*

## Results

### Ploidy Determination

For 29 species of our sample set we could derive the ploidy level from chromosome counts available in the literature (**Supplement Table S1**). To determine the ploidy level of the remaining samples, we analyzed seven species via flow cytometry. Six of them, S*. integra, S. nummularia, S. pyrenaica, S. pyrolifolia, S. rehderiana*, and *S. schwerinii*, were analyzed for the first time. All analyzed species were diploid (**Table S1**). Selected histograms are available in **Supplement Figure S1**.

### Phylogenetic Relationships

The average number of raw reads obtained from the RAD sequencing was 8.15 million reads per sample. The length after removal of adapters and barcode was 86 bp. After filtering, an average of 8.08 (+/-5.81) million reads per sample were used for clustering. An average of 161,233 clusters per sample was generated with an average depth of 52 reads per cluster. After optimization, we used a data set that consists of 23,393 RAD loci shared by at least 40 individuals and contained 320,010 variable sites of which 191,615 are parsimony informative. The concatenated alignment had a length of 1,931,205 bp with 29.06% missing data (**Supplement Table S2**). The analysis with a clustering threshold of loci shared by at least 100 individuals (m100) revealed 1,660 loci with 16,761 SNPs (**Supplement Table S2**). The amount of missing data was only 9.1%, however, the dataset was not suitable to resolve the phylogenetic relationships (see **Supplement Figure S5**). Therefore, we used this dataset only for a genetic structure analysis with STRUCTURE, which is sensitive to missing data. The HyDe results of the m40 dataset revealed 4,724 significant hybridization events of 21,420 tested combination in the taxon-assigned test based on the uSNP data. Of these, 38.6% belonged to combinations that treated a polyploid taxon as “hybrid”. The average γ-value of all observed events was 0.52.

**Figure 2:**
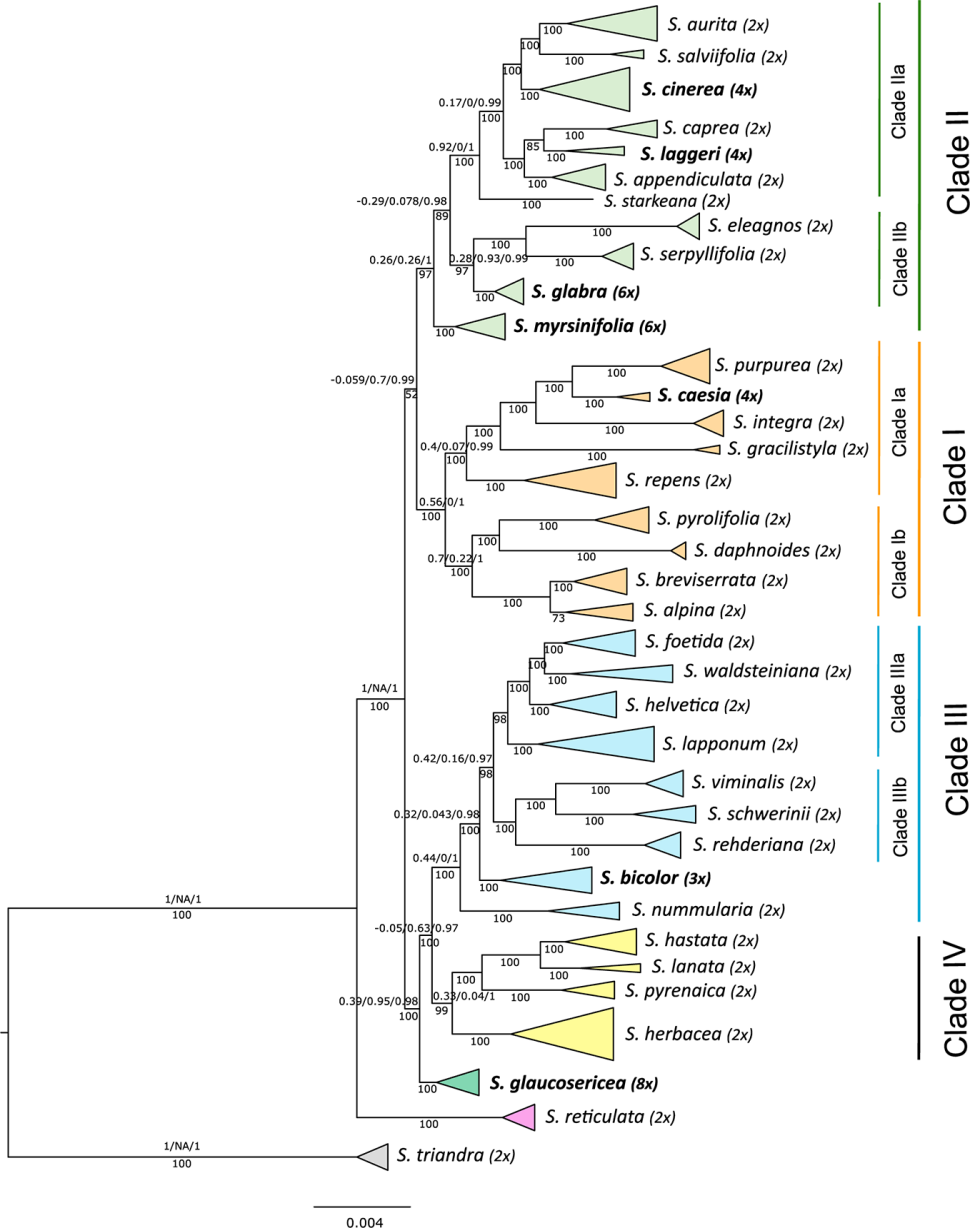
Simplified RAxML phylogeny of 133 accessions representing 35 species of *Salix* subg. *Chamaetia/Vetrix* based on 23,393 RAD loci. Four clades (numbering follows Wagner et al., 2018) are highlighted in different colours, subclades for detailed analyses are also designated. Ploidy level is indicated behind species name, polyploid samples are highlighted in bold. *Salix triandra* (subg. *Salix*) was used to root the tree. BS values below branches, QS values of selected clades and subclades above branches. A detailed phylogeny including QS support values for all branches is supplied in **Supplement Figure S2**.

The RAxML phylogeny included 133 accessions representing 35 species of the *Chamaetia/Vetrix* clade from Europe and Asia as well as four accessions of *S. triandra* as outgroup (**Figure 2**, **Supplement Figure S2**). The phylogeny revealed that all species are clearly monophyletic. The topology showed *S. reticulata* in sister position to four well-supported clades. For consistency, we used the same Roman numerals for the main clades as in Wagner et al. (2018). Clade I comprised ten species, including two well supported subclades. Clade II contained all members of section *Vetrix* as well as *S. eleagnos* and *S. serpyllifolia*, clade III comprised all members of sections *Villosae* and *Vimen* as well as triploid *S. bicolor*, and clade IV contained *S. hastata*, *S. herbacea*, *S. pyrenaica* and *S. lanata*. The tetraploid species fell into clades I and II, the triploid *S. bicolor* into clade III. The octoploid *S. glaucosericea* was in sister position to clade III and IV while hexaploid *S. glabra* was sister to *S. eleagnos* and *S. serpyllifolia* in clade II. Hexaploid *S. myrsinifolia* was in sister position to the remaining accessions of clade II. The genetic structure analysis of the complete sampling based on 1,655 uSNPs (m100) reflected the four observed main clades (I‑IV) and indicated genetic admixture of up to four clusters for the high polyploids *S. glaucosericea* and *S. myrsinifolia.* Hexaploid *S. glabra* showed admixture of “clade II” and “clade IV” (**Supplement Figure S4**).

Each clade that contained one or more polyploid samples was analyzed separately with the minimum number of accessions sharing a locus (m) dependent on the different overall number of accessions in each clade. These were m15 for clade Ia, m23 for clade II and m10 for clade III, respectively.

### Relationships of Polyploids in Clade I

**Figure 3:**
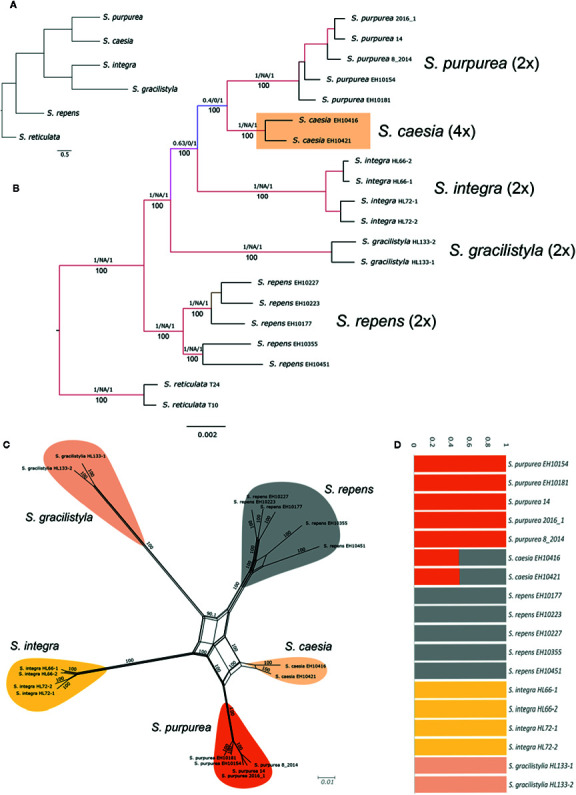
Relationships of tetraploid *S. caesia* in clade Ia. **(A)** SNAPP species tree. **(B)** RAxML phylogeny based on 38,603 RAD loci and 238,614 SNPs, respectively. Bootstrap support values below, QS values above branches. **(C)** Resulting Splitsgraph of the NeighbourNet analysis of subclade Ia. Bootstrap values indicated at branches. **(D)** Genetic structure analysis for the subclade for the most likely value of K=4 based on 37,867 unlinked SNPs.

In the RAxML phylogeny, clade I was divided into two subclades (**Figure 2**). The supported monophyletic subclade Ia (BS 100, QS 0.42/0.073/0.99) comprised the species *S. purpurea, S. caesia, S. integra* which belong to section *Helix* sensu Skvortsov (1999), as well as *S. gracilistyla.* They were in sister position to the closely related species *S. repens* (incl. *S. repens ssp. rosmarinifolia;* sect. *Incubaceae* sensu Skvortsov (1999)). Subclade Ib comprised the dwarf shrub species *S. breviserrata* and *S. alpina*, as well as the medium-sized species *S. pyrolifolia* and *S. daphnoides*. The tetraploid *S. caesia* appeared in a well-supported sister position to *S. purpurea* (BS 100, QS 0.18/0/0.99). The analysis of the 18 accessions of subclade Ia with two accessions of *S. reticulata* as outgroup based on loci shared by at least 15 accessions, contained 38,603 shared RAD loci with 238,614 SNPs. The SNAPP (**Figure 3A**) and RAxML topology (**Figure 3B**) show strong statistical support for the sister relationship of *S. purpurea* and *S. caesia*. Using 37,867 uSNPs as input and designating *S. reticulata* as outgroup, HyDe performed a test of 2,448 combinations in the individual approach of which 164 were detected as significant hybridization events. Only six of 30 tested events in the assigned taxa approach were significant. HyDe showed no significant hybridization event with *S. caesia* as hybrid. Thus, we repeated the analysis with the complete sequence data of the clade specific analysis comprising 238,614 SNPs. Here, HyDe detected 606 significant results of 2,448 tested combinations in the individual approach with an average γ-value of 0.59. In 85 hybridization events (14%) *S. caesia* was counted as “hybrid”. For these events the average observed γ-value was 0.65. The average γ-value was 0.8 in 20 significant combinations with *S. purpurea* and *S. repens* as parental species. In the m15 dataset, the 18 accessions of clade I (without outgroup) shared 35,078 RAD loci comprising 191,211 SNPs. The genetic structure analysis based on 34,071 uSNPs for the most likely K-value of four revealed genetic admixture in *S. caesia* with about 50% of each the genetic partition of *S. purpurea* and *S. repens* (**Figure 3D**). In the NeighbourNet analysis (**Figure 3C**), *S. caesia* was situated between *S. purpurea* and *S. repens*. To test a hypothesis of allopolyploid origin of *S. caesia* in more detail, we conducted a SNiPloid analysis using *S. purpurea* and *S. repens* as putative diploid parents of putative allopolyploid *S. caesia*. The results revealed 5.78% cat 1 and 12.65% cat 2 interspecific SNPs, 44.52% homeo-SNPs (cat 5), and 19.17% cat 3/4 SNPs. About 17.89% of SNPs did not fall into the given categories and were treated as “others” (**Figure 5B**).

### Relationships of Polyploids in Clade II

**Figure 4:**
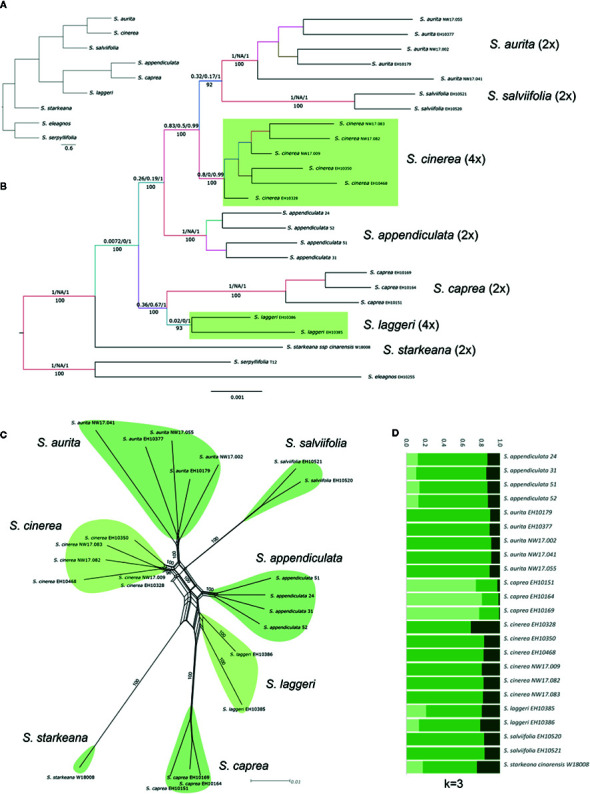
Relationships of polyploids in subclade IIa. **(A)** most abundant SNAPP species tree. **(B)** RAxML phylogeny based on 33,515 RAD loci, with QS support values above and BS values below branches Tetraploid species indicated with light green boxes. **(C)** Splitsgraph of the NeighbourNet analysis of clade IIa, BS values given at branches. **(D)** Results of the genetic structure analysis for subclade IIa (K=3) based on 39,746 unlinked SNPs.

Clade II contained hexaploid *S. myrsinifolia* and *S. glabra*, both monophyletic. The latter was in sister position to *S. eleagnos* and *S. serpyllifolia* forming subclade IIb. *Salix myrsinifolia* was in sister position to the remainders of clade II, with good BS but a skew to an alternative topology (BS 97, QS 0.28/0.93/0.99; **Figure 2**). All included members of section *Vetrix* s.l. [sensu Skvortsov (1999)] formed a well-supported monophyletic group (subclade IIa; BS 100, QS 0.92/0/1, **Figure 2**) including two tetraploid species, *S. laggeri* and *S. cinerea*. The clade-specific analysis of subclade IIa included 25 accessions and was based on 33,515 RAD loci comprising 254,819 SNPs shared by at least 23 samples (m23). The species were all monophyletic and well supported (**Figure 4B**). One accession each of *S. eleagnos* and *S. serpillifolia* served as outgroup. *Salix starkeana* was in sister position to the remaining accessions of clade IIa (BS100, 1/NA/1). The backbone of the tree topology showed some less supported branches, especially with regard to the QS values that indicate incongruencies of different branching topologies.

In the taxon-assigned approach, HyDe tested 104 combinations and yielded only four significant events. When we treated the samples as individuals, 5,313 combinations were tested and revealed 106 significant hybridization events. In 24 events (23%), *S. cinerea* was treated as ‘hybrid’. The average γ-value for these events was 0.46. For the parental combination of *S. appendiculata* and *S. aurita*, the observed nine significant hybrid combinations showed an average γ-value of 0.49. No significant hybrid event was observed for tetraploid *S. laggeri*.

**Figure 5:**
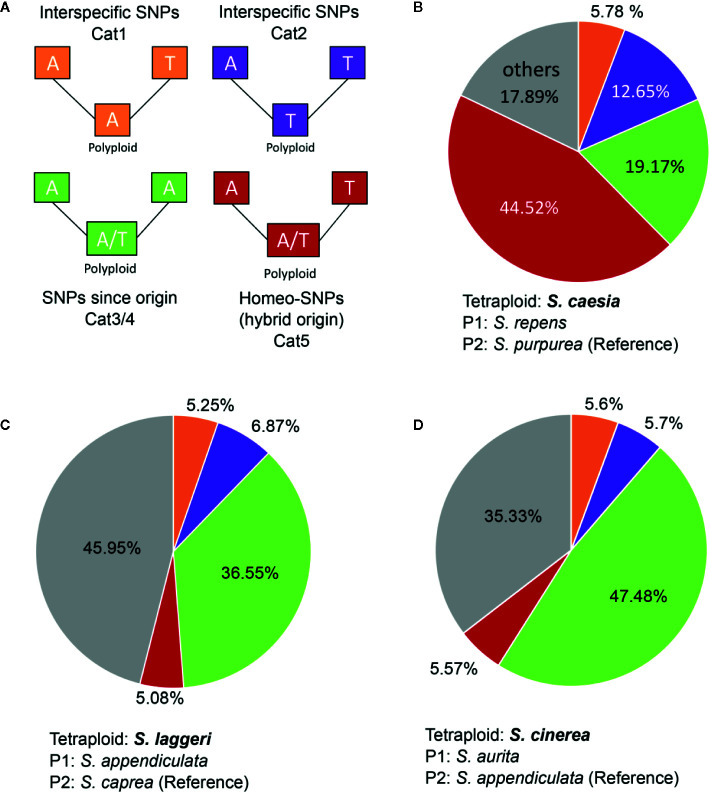
SNiPloid results for three tetraploid species. The colours in the pie diagrams represent the proportions of the different observed SNP categories: Cat1, orange, Cat2, lilac (both interspecific SNPs), Cat3/4, green (post-origin SNPs), Cat5, red (homeo-SNPs). Grey indicates the proportion of observed SNPs not falling into the five specified categories. The legend **(A)** shows an example of SNP categorization by using the same color (after Peralta et al., 2013) code. The parental samples (parent1 and parent2) are diploids. The polyploid SNPs are categorized by comparing the SNP composition with both parents. **(B)** *S. caesia*, with *S. repens* and *S. purpurea* as putative parental species, **(C)** *S. laggeri*, with *S. appendiculata* and *S. caprea* as putative parental species, and **(D)** *S. cinerea* with *S. aurita* and *S. appendiculata* as putative parental species.

The tetraploid species *S. cinerea* was in well-supported sister-position to *S. aurita* and *S. salviifolia* in both the complete and the clade specific RAxML phylogeny (**Figures 2** and **4B**), while *S. appendiculata* was sister to this clade. *Salix cinerea* was in sister position to *S. aurita* in the SNAPP species tree (**Figure 4A**) and in close relationship to *S. aurita* in the NeighbourNet analysis (**Figure 4C**). The genetic structure analysis of 39,746 uSNPs (without outgroup) for the most likely K-value of three revealed genetic admixture for all samples (**Figure 4D**). *Salix cinerea* showed a similar structure as *S. aurita* and *S. salviifolia*. The SNiPloid results for *S. cinerea* using *S. aurita* and *S. appendiculata* as potential diploid parental species, showed about 5.6% homeo-SNPs and 5.6% cat 1 and 5.7% cat 2 SNPs, respectively, whereas about 47.5% were heterozygous sites sharing one allele of only one parent (cat 3/4 SNPs); 35.3 % of SNPs did not fall into the given categories (**Figure 5D**).

Tetraploid *S. laggeri* appeared in moderately supported sister position to *S. caprea* in both the clade-specific and in the overall RAxML phylogeny (**Figures 2** and **4B**). The NeighbourNet showed *S. laggeri* in close relationship to *S. appendiculata* and *S. caprea* (**Figure 4C**). The genetic structure analysis of subclade IIa for K=3 revealed a similar genetic composition as *S. appendiculata*, with some admixture of the *S. caprea* specific partition (**Figure 4D**). The SNiPloid analysis with *S. caprea* and *S. appendiculata* as putative diploid parents revealed 5.6% homeoSNPs and about 12.2% interspecific SNPs (5.3% cat 1, 6.9% cat 2). About 36.6% of SNPs were shared with one allele of one parent (cat 3/4), while 45.9% of SNPs were not categorized (**Figure 5C**).

### Relationships of Polyploids in Clade III

Nine species grouped into clade III, which is monophyletic and well supported (BS 98, QS 0.43/0/1). The clade-specific analysis was based on 37 accessions with a minimum of 30 individuals sharing a locus and revealed 16,463 RAD loci containing 97,576 SNPs. *Salix reticulata* was used to root the RAxML tree. As illustrated in **Figure S3**, *S. nummularia* was in sister position to all remaining members of this clade followed by *S. bicolor*, which is triploid and the only polyploid species in this clade. Two subclades diverged: subclade IIIa contained the species *S. helvetica*, *S. waldsteiniana*, *S. foetida*, and *S. lapponum*, while subclade IIIb consisted of *S. viminalis*, *S. schwerinii*, and *S. rehderiana*. Each species was clearly monophyletic, and morphological sister relationships were confirmed. In the clade-specific structure analysis for the most likely K-value of five (**Supplement Figure S3**), *S. bicolor* showed genetic admixture between *S. nummularia* and subclade IIIa. The amount of admixture differed between the two samples from the Eastern Alps in Austria (about two thirds shared with subclade IIIa) and the two samples from the Harz (Brocken) in Germany (about half shared with subclade IIIa). The deep split between the two geographical regions is also reflected in the NeighbourNet network. SNiPloid was not used to analyze the putative parenthood of *S. bicolor*, for the tool is optimized for tetraploid species (containing two subgenomes) and therefore not suitable for triploid species.

## Discussion

### RAD Sequencing Data and Analysis Pipelines for Polyploids

Reduced representation libraries like RAD sequencing are frequently used to analyze intraspecific population structure, closely related species groups and to infer phylogenetic relationships of diverged lineages based on high numbers of SNPs (e.g. Cariou et al., 2013; Andrews et al., 2016; Eaton et al., 2017). However, the use of RAD sequencing for polyploid species is still hampered by the lack of suitable tools and the statistical difficulties of dealing with more than two alleles [reviewed in Clevenger et al. (2015)]. Thus, only few studies using RAD sequencing on polyploid species are published so far (Mastretta-Yanes et al., 2014; Qi et al., 2015; Brandrud et al., 2017; Feng et al., 2018; Brandrud et al., 2020). Here we present a phylogenomic study on diploid and polyploid willow species by using a de-novo assembly of RAD sequencing data.

Our assembly and concatenation of short sequenced fragments representing the whole genome resulted in a robust, fully resolved phylogeny for the *Chamaetia/Vetrix* clade. The method of concatenation has been often criticized, especially in case of conflicting signal among genomic regions. However, Rivers et al. (2016) showed that the data output of the concatenated RAD loci including thousands of unlinked SNPs is able to recover a robust tree. Our findings support the suitability of RAD sequencing for interspecific phylogenetic inference and are in accordance with many studies on different levels of divergence (e.g. Hipp et al., 2014; Eaton et al., 2017; Wagner et al., 2018; He et al., 2020). The allelic information of the polyploid samples can be condensed to a single consensus sequence to circumvent the challenges of dealing with more than two alleles for phylogenetic approaches. However, the loss of information caused by this simplification might lead to wrong placement of the polyploid accessions in the phylogeny (Eriksson et al., 2018; Andermann et al., 2019). Our results showed that the RAD sequencing data contain enough information to resolve the phylogenetic relationships in *Salix* and that condensed allelic information was appropriate for the included allopolyploid species. While for phylogenetic approaches complete sequence information is preferable (Leaché et al., 2015), many unlinked loci are the preferable source of information for analyses, where the sites are treated as independently evolving genetic entities. Thus, we used the unlinked SNPs for network and genetic structure analyses. The uSNPs were also successfully used to reconstruct the species tree of each subclade using a coalescence-based method implemented in SNAPP (Bryant et al., 2012). Our results confirmed that the coalescent approach using RAD sequencing data is suitable for species delimitation, as recently shown by Brandrud et al. (2020).

HyDe (Blischak et al., 2018a) is a tool to detect hybridization and we showed that it is possible to use it with RAD sequencing data. We used HyDe to draw conclusions on the putative parental species of allopolyploids. First, we used the uSNP data, as suggested by Blischak et al. (2018a). The complete alignment using all SNPs, however, revealed a higher amount of significant results. Therewith we confirm the findings of Blischak et al. (2018a) that the number of significant hybridization events depends on the size of the input data and more input data reveal more accurate results. HyDe detected 4,724 significant hybridization events in the complete dataset. About 40% of them were combinations including a polyploid as “hybrid”. That means that 60% were indicated as hybridization events between diploid samples. The high amount of natural hybridization in *Salix* is well known (Skvortsov, 1999; Argus, 2010), and homoploid hybridization even between distantly related species has been documented (Hardig et al., 2000; Gramlich et al., 2018). However, in this study we observe clearly distinct monophyletic groups for all included species and the sampled accessions represent morphologically definite individuals. We assume that HyDe uncovers a high amount of ancient gene flow that resulted in a shared polymorphism in the whole phylogeny and its subclades. We further suppose that non-visible introgression or, that the huge amount of input data cause significant number of “false” hybrid combinations.

SNiPloid (Peralta et al., 2013) was used to reconstruct the genomic constitution of the tetraploid species. SNiPloid was originally developed for transcriptomic data (Peralta et al., 2013). The application of this tool to RAD sequencing data provided us valuable information about the contributions of putative parental species to the tetraploid genome. In combination with genetic structure, HyDe and NeighborNet analyses, we could test for allopolyploid origin and revealed some insights into genome evolution after the polyploidization events. Unfortunately, the tool is only suitable for tetraploid species that consist of not more than two subgenomes (Peralta et al., 2013). However, for RAD sequencing data SNiPloid provides an alternative approach by using biallelic SNPs instead of sequence data to reveal potential parenthood of allotetraploids without previous knowledge. For the interpretation of the SNiPloid data it must be considered that in most cases, we did not test the “real” parental accessions. Our results are instead based on putative parental species/lineages, and we therefore expect a certain amount of SNPs that do not fall into the narrowly defined SNP categories. This will lead to an increase of proportions of non-categorized “other” SNPs. Furthermore, proportions of SNP categories must be interpreted with caution as also the intra-specific sampling can influence the actual amounts of detected SNPs for the respective categories. Nevertheless, we can present here a first overview of the origin and evolution of polyploid species within a phylogenetic framework. This increases the potential to analyze polyploid samples with reduced representation methods.

### Phylogenetic Relationships and Origins of Polyploids in *Salix*

The RAD sequencing data revealed a well-resolved phylogeny of the *Chamaetia/Vetrix* clade including 35 Eurasian species plus *S. triandra* as outgroup and thus provide a sufficient framework for reconstructing the origin of the polyploid species. The presented phylogeny included members of all sections sensu Skvortsov (1999) and hence covered the morphological diversity and biogeographical range of Eurasian willows. All of the 36 included species are monophyletic. The isolated position of *S. reticulata* and the observed four clades are in accordance with a former study on European diploid species by Wagner et al. (2018). The samples from Asia fall into the four clades.

About 40% of *Salix* species are polyploid (Suda and Argus, 1968) ranging from tetraploid to octoploid, rarely to decaploid. Our flow cytometry results showed a diploid level for the six included species from Asia that were previously not investigated (**Supplement Table 1**, **Supplement Figure S1**). Although some studies are published about tetraploid species in subgenus *Salix* s.l. (Triest et al., 2000; Triest, 2001; Barcaccia et al., 2014) no molecular studies existed so far on the origin of polyploid species of the *Chamaetia/Vetrix* clade. In this study we included seven polyploids from Europe with different ploidy levels to study different scenarios of their evolutionary origin. For the polyploid species included here, no diploid cytotypes have ever been reported, and they all exhibit very distinct morphologies and a genetic structure that is composed of several genetic clusters. These features make autopolyploid origins unlikely. The included polyploid species are all monophyletic. However, the species appear scattered over the phylogeny, indicating multiple independent origins of polyploids within the genus resulting from different parental combinations. Thereby *Salix* differs from plant genera in which a single allopolyploidization event resulted in post-origin adaptive radiation and speciation (e.g., the Hawaiian Silversword alliance, Seehausen, 2004). Willows rather resemble plant genera with repeated independent polyploidization events, like in *Nicotiana* (Chase et al., 2003), *Achillea* (Guo et al., 2013), *Ranunculus* (Baltisberger and Hörandl, 2016), and *Dactylorhiza* (Brandrud et al., 2020), among others.

The higher polyploid willows are in sister position to major clades or on basal branches. This could be explained by an old allopolyploid origin of these species. We would expect this pattern if two or more ancestral lineages of the extant major subclades (I-IV) had contributed to the polyploid genome. However, these ancestral lineages are probably not conspecific to any of the extant species. According to the crown group age of the *Chamaetia/Vetrix* clade (23.76 Ma, Wu et al., 2015), their origins may date back to the late Miocene/ early Pliocene. However, next to that, the hybrid nature of their origin might be responsible for the placement of the polyploids on basal branches of the subclades in the phylogeny. Our structure analyses indicated that the hexa- and octopolyploids, but also triploid *S. bicolor*, harbor two to four genetic partitions that characterize otherwise the main subclades I-IV (see **Figure S4**). Hence, we assume allopolyploid origins from divergent lineages. However, since the genetic structure analyses cannot disentangle ancient and recent hybridization or introgression events, further studies on the origin on the high polyploid willows are necessary.

### Origin and Evolution of the Allotetraploid Species

The tetraploids fall into the observed clades (**Figure 2**). Since all included tetraploid species are monophyletic and do form well-supported entities in our analysis, we do not assume multiple origins with different parental combinations. Instead, we assume a single or few hybrid (allopolyploid) origin(s) from a pool of related individuals (Abbott et al., 2013), followed by speciation. We are aware that extant species might not represent the true parental species, and that extinct taxa might have been also involved. Nevertheless, by testing different combinations of putative diploid parents that are the next extant relatives in our observed trees, we analyzed potential parental lineages that might have contributed to allotetraploid species formation. The comparison of the two respective subclades, however, suggests different evolutionary scenarios.

Clade Ia comprised species assigned to *Salix* sect. *Helix* sensu Skvortsov (1999), except for *S. gracilistyla*. However, all these species share the morphological character of connate filaments. The SNAPP species tree revealed a close relationship of *S. caesia* and S*. purpurea*, supporting the RAxML analyses. Based on our genetic structure analyses, tetraploid *S. caesia* combined the genetic partitions that occur in *S. purpurea* and *S. repens*, with about equal proportions, while the two Asian species *S. gracilistyla* and *S. integra* showed each a different genetic partition. The NeighbourNet analysis confirmed these findings and placed *S. caesia* between the *S. purpurea* and *S. repens* (**Figure 3C**). These two species are sympatric with *S. caesia* (Skvortsov, 1999). HyDe detected 14% significant hybridization events for *S. caesia* based on the complete sequence data. In case of a comparatively young hybridization event we would expect γ-values between 0.4 and 0.6. The high observed average γ-value of 0.8 for the combinations of *S. purpurea* and *S. repens* supports the hypothesis of an older event or other processes like incomplete lineage sorting. In so far, the HyDe analysis corroborates results of our SNiPloid analysis that revealed a high proportion of homeo-SNPs (44.52%) derived from the two parental species, whereas proportions of SNPs from post-origin interspecific events were much lower (18.53% for cat 1+2). However, the observed amounts of interspecific SNPs (cat1, cat2) may also be the result of incomplete lineage sorting. Thus, the observed results support the hypothesis of an allotetraploid origin from *S. purpurea* and *S. repens* s.l., with rather few post-origin backcrossing events. Indeed, no extant hybrids of *S. caesia* with the putative parental species have been reported so far, and hybridization with other species is extremely rare (Hörandl et al., 2012). The considerable proportion of post-origin SNPs (Cat 3/4, 19.17%) specific for the polyploid *S. caesia* indicates an independent evolution of the lineage. Occupation of niches at higher elevations may have contributed to reproductive isolation of *S. caesia* from the two parental species that occur in lowlands (Hörandl et al., 2012).

The tetraploid species *S. cinerea* and *S. laggeri* belong to subclade IIa that contain all included species of section *Vetrix* sensu Skvortsov (1999). The members of this section are medium sized shrubs with hairy leaves occurring from lowland to montane regions. They grow often in sympatry with other species in mixed populations and hybridize frequently, also with species of other sections (Hörandl et al., 2012). A big, shared gene pool is visible in high proportions of “other”, unassigned SNPs in SNiPloid. High ongoing gene flow may explain that the clade-specific genetic structure analysis revealed no clear species-specific partitions within subclade IIa. However, both *Salix laggeri* and *S. cinerea* showed high proportions of species-specific SNPs (36.6% and 47.5%, respectively), indicating independent evolutionary lineages. The most likely parental combinations for their origin revealed in both cases around 5% of homeo-SNPs derived from hybrid origin, and also low proportions of post-origin hybridization with both parents, supporting allopolyploid origin. We cannot rule out that the true parental lineages were not included here – either due to extinction or to involvement of species that occur nowadays outside Europe (e.g., in adjacent Russia). A long evolutionary history with post-origin hybridization/ introgression events further affected the genomic composition of the hybrids. Interestingly, the two tetraploids differ strongly in the results of HyDe analyses: *S. cinerea* is responsible for 23% of significant hybridization events in the range of a more recent hybrid, while no significant hybrid event including *S. laggeri* was detected. Extant hybridization of *S. cinerea* with other co-occurring lowland species (*S. aurita, S. caprea, S. eleagnos, S. myrsinifolia, S. repens*, and a hybrid series with *S. viminalis*, Hörandl et al., 2012) may explain this result, while *S. laggeri* is a subalpine species that is reproductively better isolated from the other species and hybridizes only occasionally with the subalpine, sympatric *S. appendiculata* (Lautenschlager-Fleury and Lautenschlager-Fleury, 1986; Hörandl et al., 2012).

Our results on tetraploids confirmed an allopolyploid origin and a dynamic post-origin evolution of genomes, indicating speciation and evolution of independent lineages. Distribution ranges and ecological niches of the parental species, however, could have fluctuated from the origin of the clade onwards and may have caused various secondary contact hybridizations in different time periods of the Cenozoic (Hewitt, 2004; Hewitt, 2011). The relatively high age of the whole *Chamaetia/Vetrix* clade with a crown group age in the late Miocene, and the lack of a dated phylogeny makes it difficult to pinpoint hybridization events to certain geological time periods. According to extant hybridization patterns, isolation of the polyploid willows appears to be strongly influenced by the strength of habitat differentiation as discussed by Martini and Paiero (1988); Hörandl et al. (2012) and, Gramlich et al. (2016) for Central European species, and by Huang et al. (2015) on species from Taiwan. These findings support the notion that occupation of a separate niche is important for the establishment of a newly formed polyploid willow lineage.

## Conclusions

Our data demonstrate that high-quality RAD sequencing data allow for the reconstruction of phylogenetic frameworks and give insights into origin and evolution of polyploid species. In willows, polyploidization appears to be predominantly connected to hybridization, i.e. to allopolyploid origin, as hypothesized by Skvortsov (1999). Our data suggest that polyploids harbor considerable proportions of lineage-specific SNPs and managed to establish stable, self-standing evolutionary lineages after allopolyploid origin.

## Data Availability Statement

The datasets presented in this study can be found in online repositories. The names of the repository/repositories and accession number(s) can be found below: https://www.ncbi.nlm.nih.gov/, PRJNA433286.

## Author Contributions

NW and EH planned and designed research. NW, LH and EH conducted fieldwork. NW and LH performed experiments, NW analyzed data, and established new pipelines. NW and EH wrote the manuscript with contributions from LH.

## Funding

This study was financially supported by the German research foundation DFG (Ho 5462/7-1 to EH), the National Natural Science Foundation of China (31800466 to LH), and the Natural Science Foundation of Fujian Province of China (2018J01613 to LH). LH was sponsored by China Scholarship Council for his research stay at the University of Goettingen (201707870015).

## Conflict of Interest

The authors declare that the research was conducted in the absence of any commercial or financial relationships that could be construed as a potential conflict of interest.
