# Supplementary material for: Phylogenomic Relationships and Evolution of Polyploid Salix Species Revealed by RAD Sequencing Data
Source: Front Plant Sci. 2020 Jul 17;11:1077. doi: 10.3389/fpls.2020.01077 (PMC7379873; doi:10.3389/fpls.2020.01077)
Supplement: Supplementary file 1 [file Image_1.pdf]

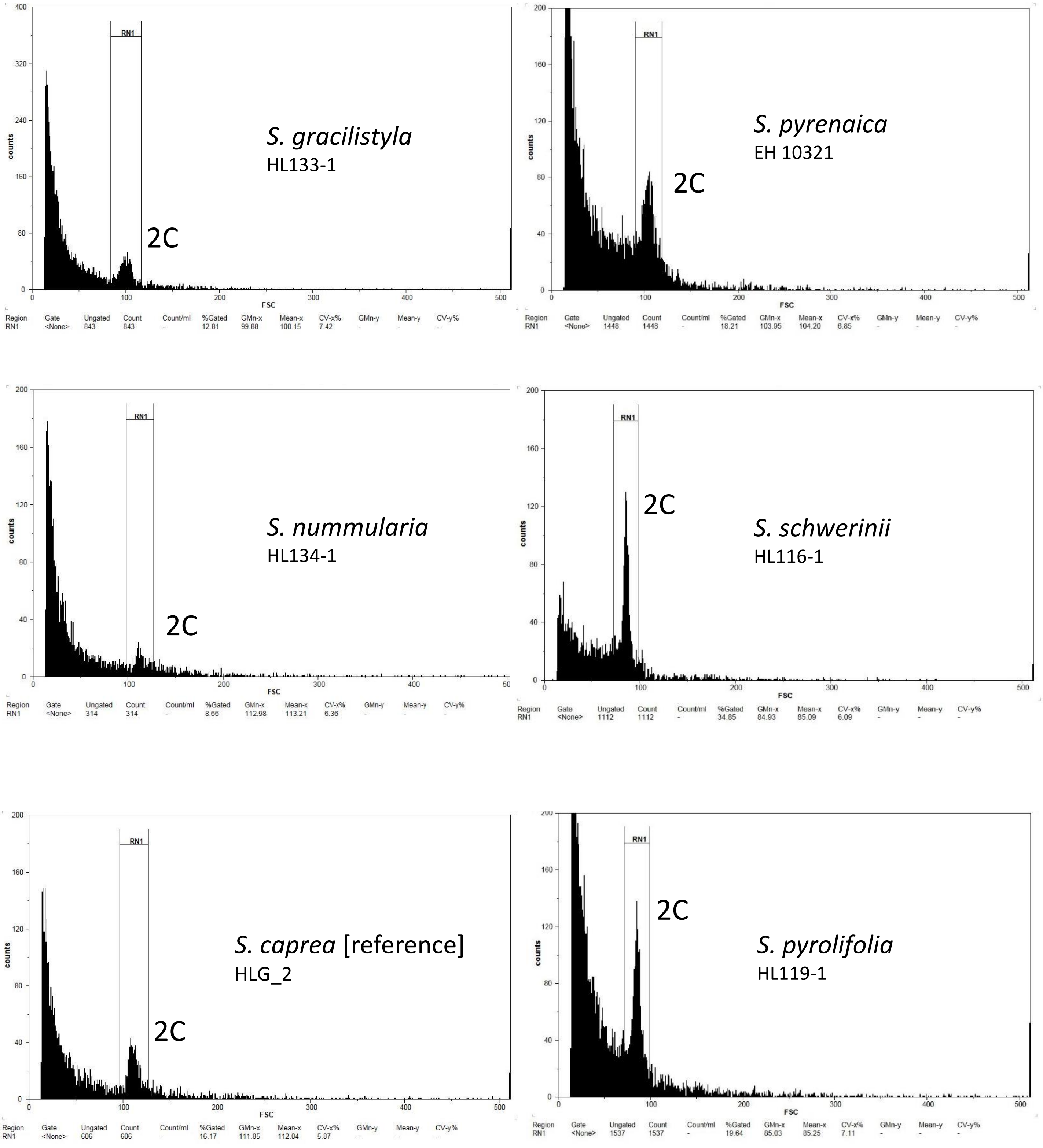

Supplement Fig. S1: selected flow cytometry histograms for measurements of included *Salix* species. Diploid *Salix caprea* was used as an external reference.
