# Supplementary figures and images for: Phylogenomic Relationships and Evolution of Polyploid Salix Species Revealed by RAD Sequencing Data
Source: Front Plant Sci. 2020 Jul 17;11:1077. doi: 10.3389/fpls.2020.01077 (PMC7379873; doi:10.3389/fpls.2020.01077)

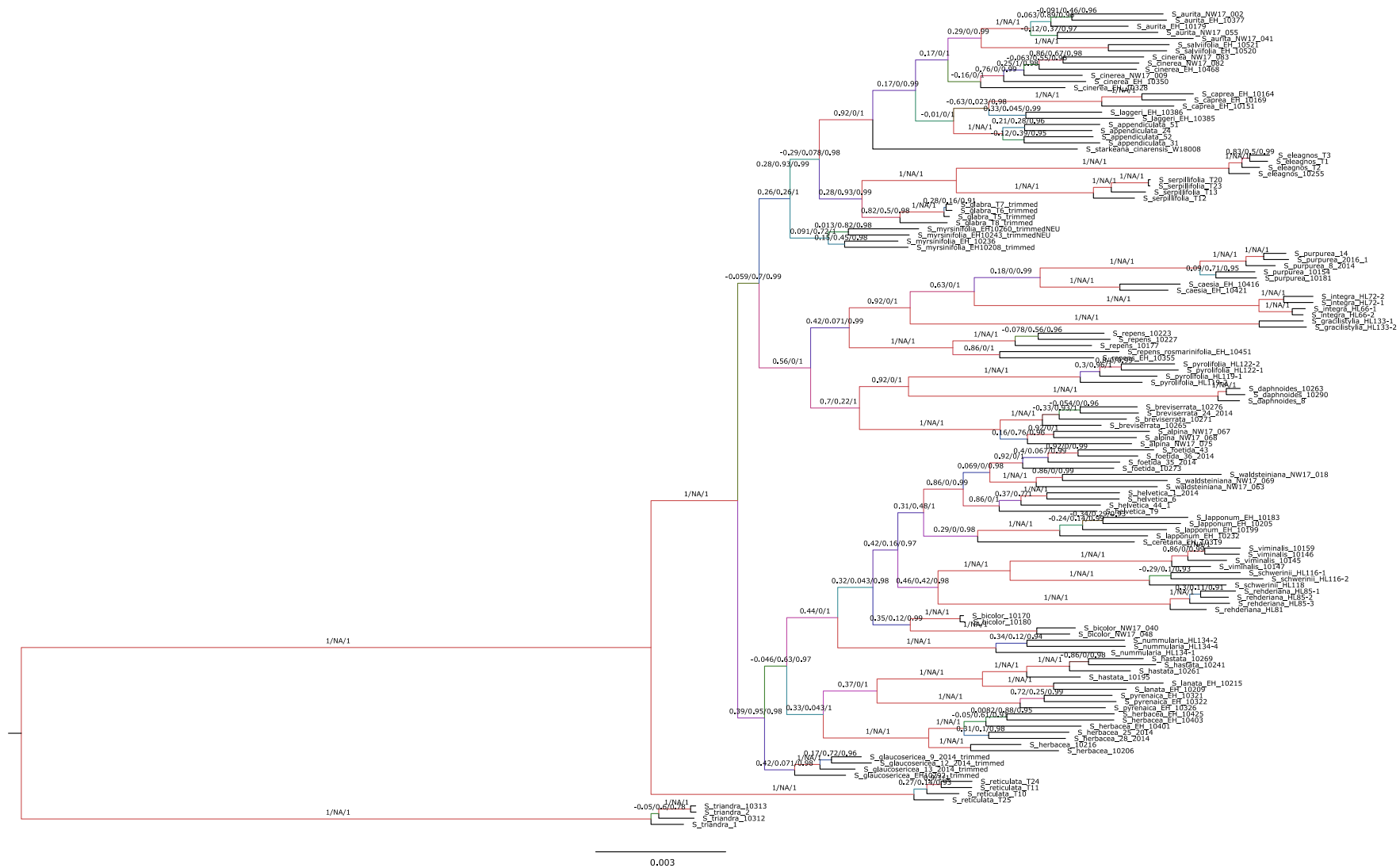

Supplement: Supplementary file 2 [file Image_2.pdf]
