# Supplementary material for: Phylogenomic Relationships and Evolution of Polyploid Salix Species Revealed by RAD Sequencing Data
Source: Front Plant Sci. 2020 Jul 17;11:1077. doi: 10.3389/fpls.2020.01077 (PMC7379873; doi:10.3389/fpls.2020.01077)
Supplement: Supplementary file 4 [file Image_4.pdf]

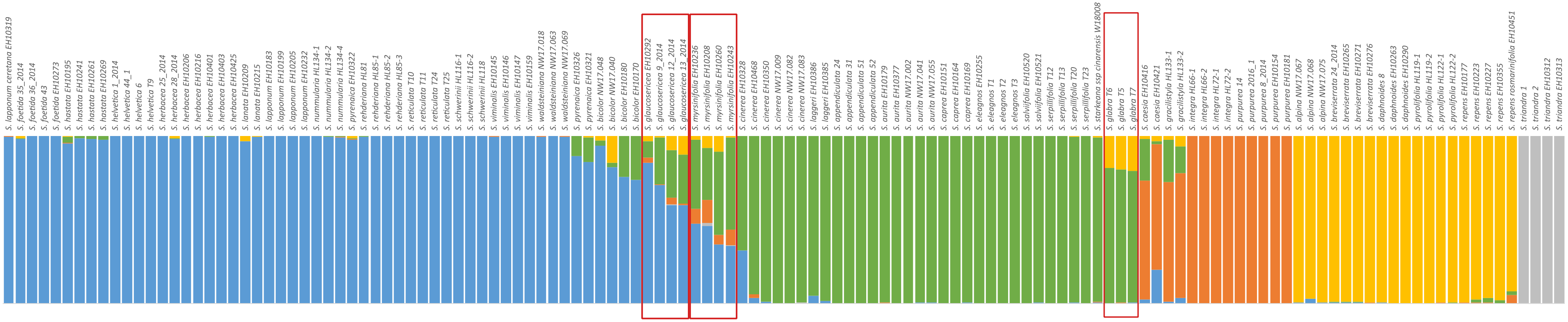

Supplement Fig. S4. Genetic structure analysis of the complete data set including 133 taxa samples based on 1,655 unlinked SNPs for most likely K=5. Higher polyploids *S. glaucosericea* (8x), *S. myrsinifolia* (6x) and *S. glabra* (6x) are indicated by red boxes.
