# Supplementary material for: Phylogenomic Relationships and Evolution of Polyploid Salix Species Revealed by RAD Sequencing Data
Source: Front Plant Sci. 2020 Jul 17;11:1077. doi: 10.3389/fpls.2020.01077 (PMC7379873; doi:10.3389/fpls.2020.01077)
Supplement: Supplementary file 5 [file Image_5.pdf]

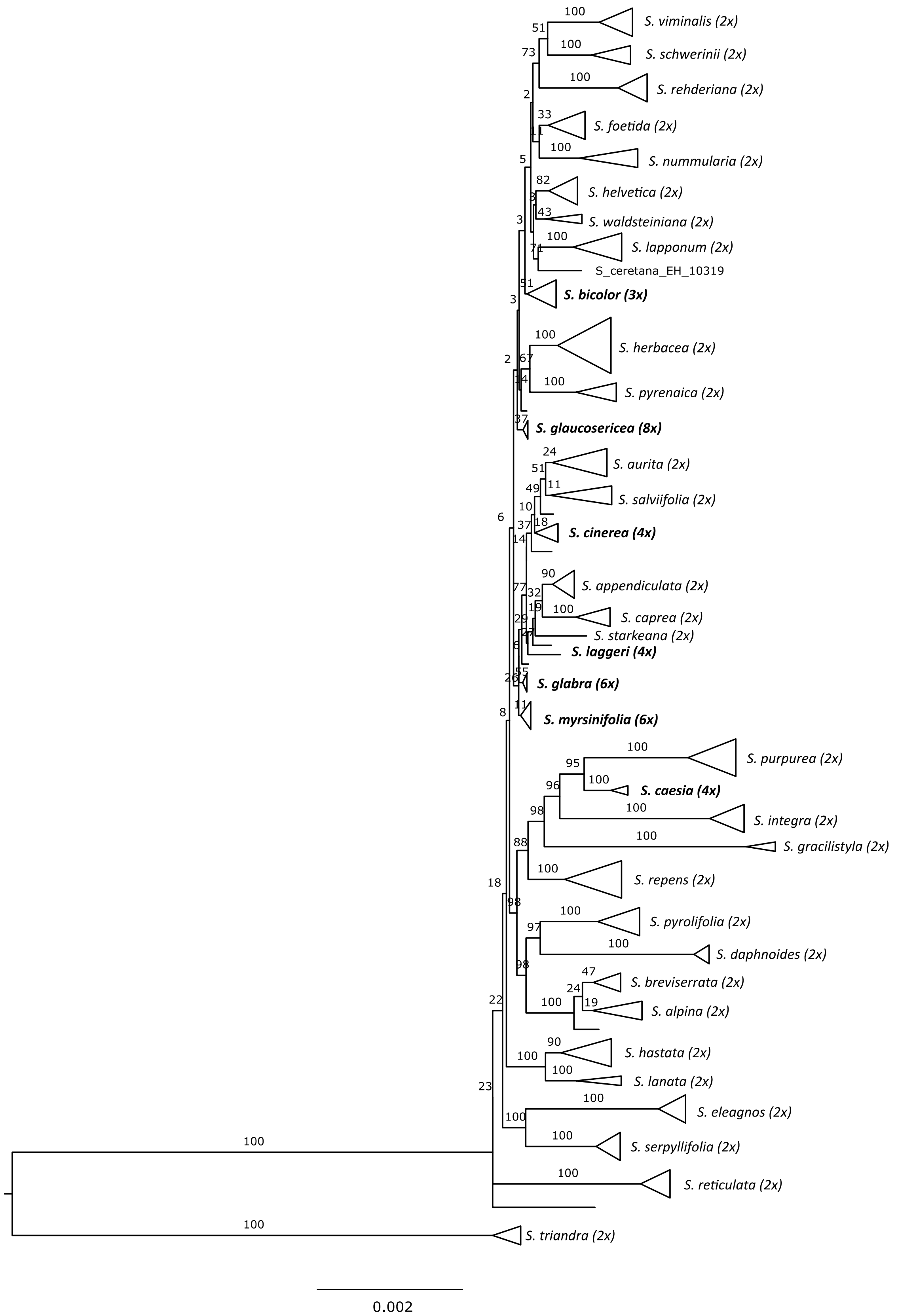

Fig. S5. Simplified RAxML phylogeny of 133 accessions representing 35 species of *Salix* subg. *Chamaetia/Vetrix* based on 1,660 RAD loci resulting from the m100 dataset. Ploidy level is indicated behind species name, polyploid samples are highlighted in bold. *Salix triandra* (subg. *Salix*) was used to root the tree. BS values above branches.
