# Supplementary material for: Phylogenomic Relationships and Evolution of Polyploid Salix Species Revealed by RAD Sequencing Data
Source: Front Plant Sci. 2020 Jul 17;11:1077. doi: 10.3389/fpls.2020.01077 (PMC7379873; doi:10.3389/fpls.2020.01077)
Supplement: Supplementary file 7 [file Table_2.pdf]

Table S2. **Statistics for tested ipyrad parameter sets for parameter optimization for 133 taxa.**

Main settings: Within and among sample clustering threshold of 85% similarity, min. read length 35bp, min depth for SNP base calling: six, maximum of two alleles per locus, max. 20 SNPs per locus, max. 8 indels per locus.

minimum number of taxa per locus	number of loci	number of SNPs	number of PIS
4	125,338	579,784	264,298
20	14,580	125,029	71,213
40	4,547	46,042	25,745
80	1,948	19,747	10,501
100	1,660	16,761	8,921
120	1,259	12,554	6,666

Main settings: Within and among sample clustering threshold of 85% similarity, min. read length 35bp, min depth for SNP base calling: six, maximum of four alleles per locus, max. 20 SNPs per locus, max. 8 indels per locus.

minimum number of taxa per locus	number of loci	number of SNPs	number of PIS
20	14,580	461,863	273,811
40	23,393	320,010	191,615

Average statistics for final m40 dataset, average numbers and standard derivation**.**

	# raw reads per sample in million	# filtered reads per sample in million	# clusters per sample	read depth per cluster	# loci per sample in assembly
average (+/-)	8.08 +/- 5.81	8.02 +/-5.66	161,233 +/-88,738	51.94 +/- 28.75	16,938 +/- 2,312
