# Supplementary material for: Phylogenomic Relationships and Evolution of Polyploid Salix Species Revealed by RAD Sequencing Data
Source: Front Plant Sci. 2020 Jul 17;11:1077. doi: 10.3389/fpls.2020.01077 (PMC7379873; doi:10.3389/fpls.2020.01077)
Supplement: Supplementary file 3 [file Image_3.pdf]

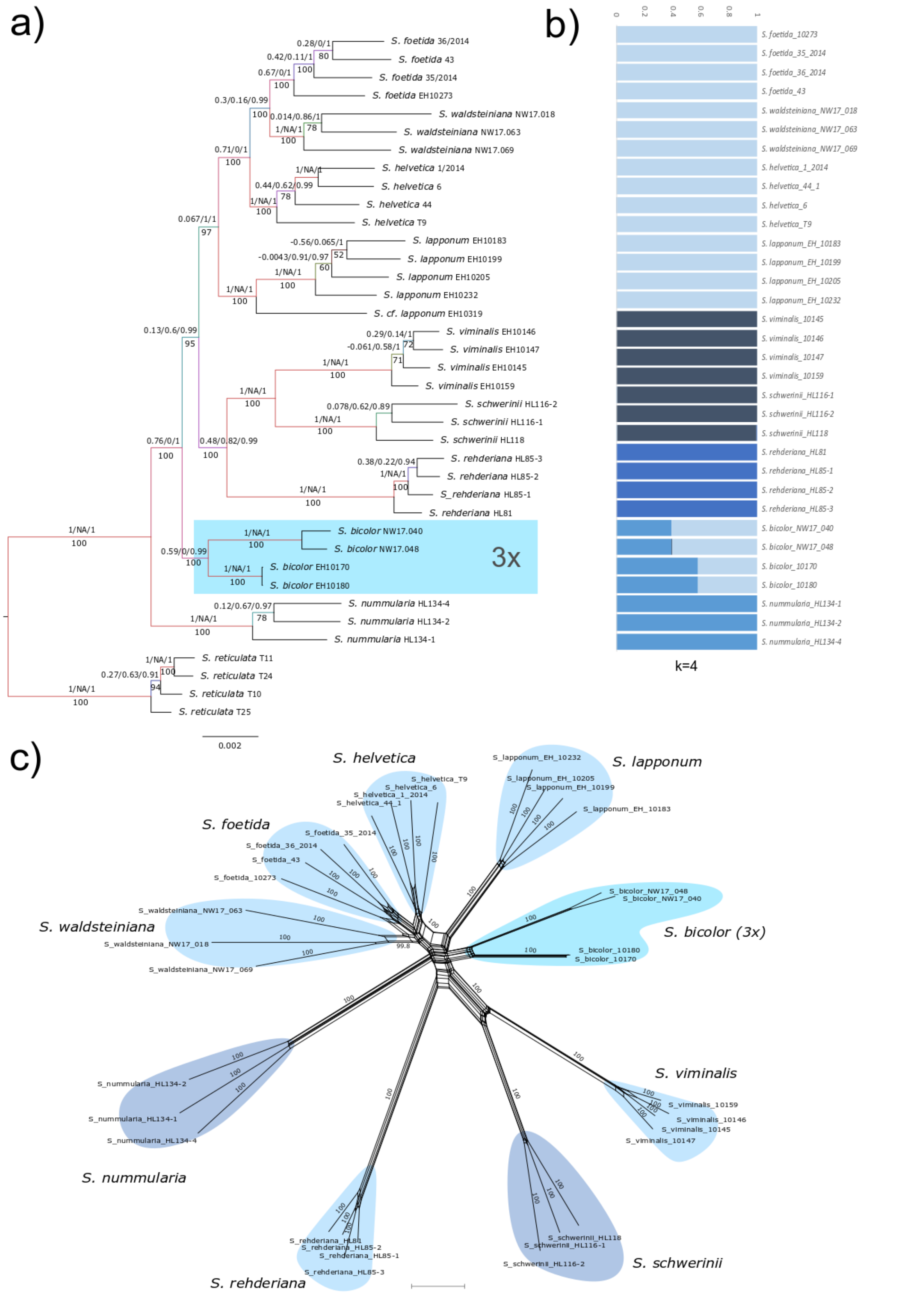

Fig. S3. Relationships of triploid *S. bicolor* in clade III. a) RAxML phylogeny based on 16,463 concatenated RAD loci and 97,576 SNPs, respectively. *S. reticulata* was used as outgroup to root the tree. Bootstrap support values below and QS support values above branches. Triploid S. bicolor is highlighted in a blue boxb) Genetic structure analyses based on 16,104 unlinked SNPs for most likely K=4. c) Splitsgraph of the NeighbourNet analysis of clade III, bootstrap values given at branches.
